# Gamma oscillations in V1 are correlated with GABA_A_ receptor density: A multi-modal MEG and Flumazenil-PET study

**DOI:** 10.1038/srep16347

**Published:** 2015-11-17

**Authors:** Jan Kujala, Julien Jung, Sandrine Bouvard, Françoise Lecaignard, Amélie Lothe, Romain Bouet, Carolina Ciumas, Philippe Ryvlin, Karim Jerbi

**Affiliations:** 1 Department of Neuroscience and Biomedical Engineering, Aalto University, 02150 Espoo, Finland; 2 Lyon Neuroscience Research Center, INSERM U1028—CNRS UMR5292, F-69000, Lyon, France; 3 Department of Epileptology and Functional Neurology, Lyon Neurological Hospital, F-69000, Lyon, France; 4 CERMEP imaging center, F-69003, Bron, France; 5 Institute for Child and Adolescent with Epilepsy (IDEE), F-69000, Lyon, France; 6 Department of Clinical Neurosciences, CHUV, 1011, Lausanne, Switzerland; 7 Department of Psychology, University of Montreal, H3C 3J7 Montreal, Québec, Canada

## Abstract

High-frequency oscillations in the gamma-band reflect rhythmic synchronization of spike timing in active neural networks. The modulation of gamma oscillations is a widely established mechanism in a variety of neurobiological processes, yet its neurochemical basis is not fully understood. Modeling, *in-vitro* and *in-vivo* animal studies suggest that gamma oscillation properties depend on GABAergic inhibition. In humans, search for evidence linking total GABA concentration to gamma oscillations has led to promising -but also to partly diverging- observations. Here, we provide the first evidence of a direct relationship between the density of GABA_A_ receptors and gamma oscillatory gamma responses in human primary visual cortex (V1). By combining Flumazenil-PET (to measure resting-levels of GABA_A_ receptor density) and MEG (to measure visually-induced gamma oscillations), we found that GABA_A_ receptor densities correlated positively with the frequency and negatively with amplitude of visually-induced gamma oscillations in V1. Our findings demonstrate that gamma-band response profiles of primary visual cortex across healthy individuals are shaped by GABA_A_-receptor-mediated inhibitory neurotransmission. These results bridge the gap with *in-vitro* and animal studies and may have future clinical implications given that altered GABAergic function, including dysregulation of GABA_A_ receptors, has been related to psychiatric disorders including schizophrenia and depression.

Neuronal oscillations across multiple frequencies are a prominent feature of brain function. In particular, high-frequency neural oscillations and broadband activity in the gamma-band (ca. 30–200 Hz) have been demonstrated to be involved in a wide range of primary and high-level cognitive processes^1^,^2^,^3^,^4^,^5^,^6^,^7^,^8^,^9^,^10^,^11^. Interestingly, the blood oxygen level dependent signal (BOLD) used in functional magnetic resonance imaging has also been shown to correlate with the gamma-band activity^12^, across a variety of brain regions^13^,^14^.

Yet, despite being the subject of a thriving field of research, the neurochemical mechanisms underlying the emergence of gamma-band oscillations in the human brain remain poorly understood. Evidence from animal studies suggests that they are generated by a neuronal network containing interconnected pyramidal cells and GABA (gamma-aminobutyric acid)-ergic inhibitory interneurons^15^,^16^. In line with this, gamma-oscillations measured with Magnetoencephalography (MEG) have been shown to depend on GABA concentration levels estimated via magnetic resonance spectroscopy (MRS) in primary visual^17^,^18^, primary motor^19^, left dorsolateral prefrontal cortex^20^. However, MRS-based GABA-Gamma correlations are challenged, to a certain extent, by partly inconsistent findings^21^,^22^,^23^, as well as failure to replicate the correlation between GABA-concentration and gamma oscillation frequencies in V1^24^. These diverging results could be due to the fact that MRS GABA concentration does not specifically quantify synaptic GABA and that it is susceptible to macromolecule contamination^24^. Other more specific markers of GABAergic synaptic signaling might thus be necessary to reliably probe its putative link to gamma oscillations.

We hypothesized that GABA_A_ receptor density, which is more closely related to inhibitory neurotransmission than total GABA concentration, could provide a more direct assessment of the relationship between GABAergic signaling and gamma oscillations in humans. Recent findings in rodents have shown that high-frequency oscillations in the ripple range (80–250) are abolished following focal GABA_A_ receptor blockade^25^. Furthermore, in a mouse model of schizophrenia, the disruption of GABA_A_ receptor subunit clustering has been associated with a reduction of gamma power in the ca. 40–70 Hz range^26^. In addition, ample evidence from modeling and *in-vitro* studies point to the key role of GABA_A_ receptor mediated inhibition in the generation of gamma oscillations^8^,^16^,^27^,^28^. Reliable evidence for such a link in humans is still missing.

We set out to evaluate the effects of variations in GABA_A_ receptor distribution, estimated during rest, on rhythmic activity recorded during visual perception. To this end, we investigated the relationships between the timing, amplitude and frequency of alpha-, beta- and gamma-band oscillatory activity (determined with MEG) and GABA_A_ receptor density (determined with ^11^C-flumazenil positron emission tomography, FMZ-PET) in primary visual cortex (V1). We hypothesized that the PET-based GABA_A_ receptor measure would form an informative proxy for the strength of inhibition in the V1 neural network, and, thus, predict the properties of visual gamma-band oscillations across individuals. Our results demonstrate that the density of GABA_A_ receptors correlates positively with gamma peak frequency and negatively with gamma amplitude in human primary visual cortex.

## Results

To investigate the relationship between GABAergic inhibition and high-frequency oscillations in V1, we measured GABA_A_ receptor density using FMZ-PET and gamma oscillation dynamics with MEG in the same population of healthy individuals (n = 13, age 19–29 yrs, mean 24 yrs). The FMZ-PET data used to determine the GABA_A_ receptor density were collected while the subjects were resting in the scanner, and the characteristics of oscillatory neuromagnetic signals in each individual were estimated from their visual responses in a standard n-back working memory task.

### Significant modulation of alpha-, beta and gamma-band activity and high density of GABA_A_ receptors in V1

**Figure 1:**
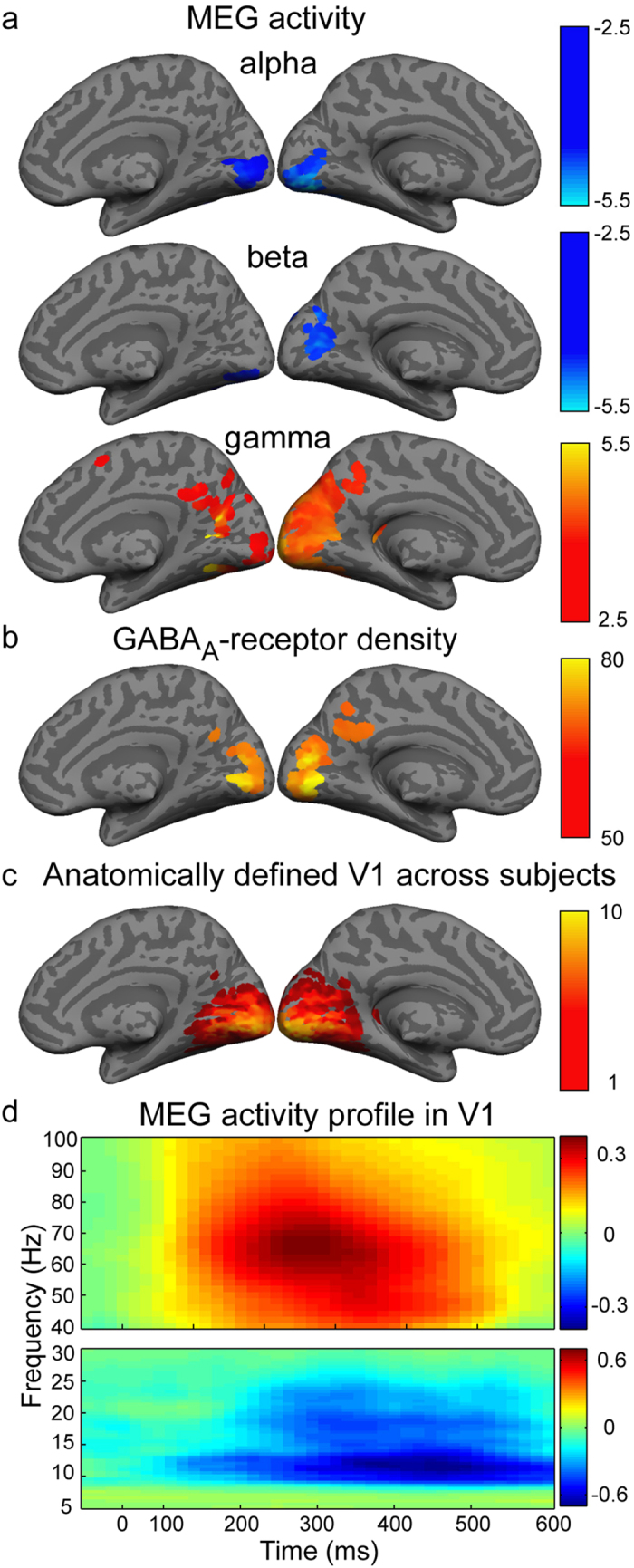
Medial views of modulation of neural activity, distribution of GABA_A_ receptors and the primary visual cortex (V1). (**a**) Modulation of neural activity in the alpha-, beta- and gamma-bands with respect to baseline time-window (paired t-test, p < 0.005, uncorrected). (**b**) Cortical GABA_A_ receptor density across subjects (threshold at mean + 1 SD). (**c**) Consistency of V1 in the template brain across subjects. (**d**) Group-level neural responses (normalized units) in the anatomically defined bilateral V1. The data are shown for the 10 subjects for whom both MEG and PET data were recorded successfully. Cortical-level visualization was performed using FreeSurfer software^57^.

**Figure 2:**
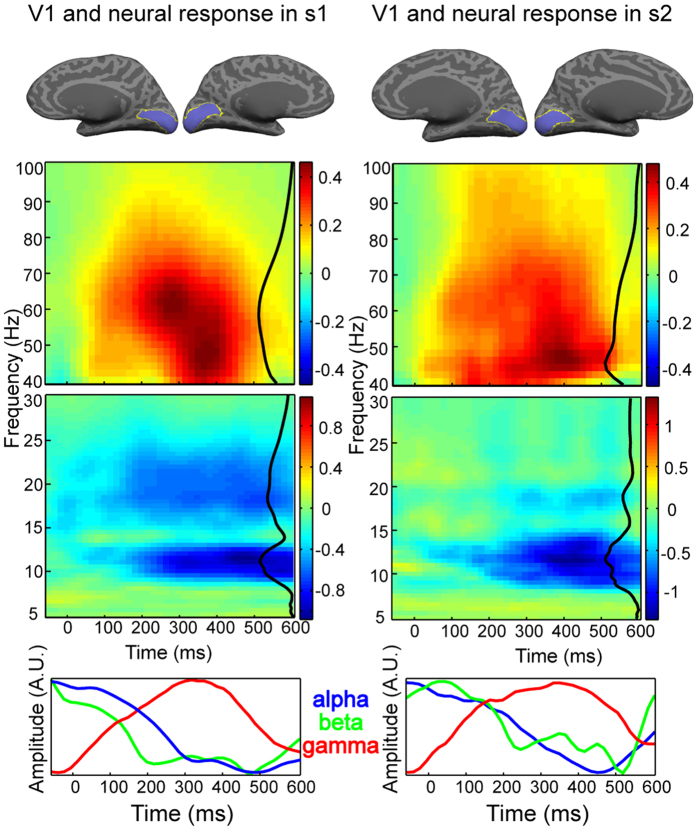
Anatomical definition of and the neural responses in V1 for 2 subjects. Anatomically defined V1 (top), time-frequency representations (TFR) of the neural responses (middle), and the time-courses of modulation of activity in the alpha-, beta, and gamma-bands (bottom). In the TFRs, the black lines show the spectral behavior across the 0 to 600 ms time-window. In the lower bands (5–30 Hz), the curves have been inverted (prominent deflection indicates strong suppression). Cortical-level visualization was performed using FreeSurfer software^57^.

Visual stimulation at task onset induced a significant (p < 0.005) prominent gamma-band response within a window of 0–600 ms in bilateral occipital and occipito-temporal cortices (Fig. 1a). In parallel, both alpha and beta bands showed significant (p < 0.005) suppression of activity with respect to baseline (-200 to 0 ms) in medial occipital cortex (Fig. 1a). No significant modulation of activity was detected in the theta-band (4-7 Hz) at the selected threshold (p < 0.005). The highest density of GABA_A_ receptors across the cortex was evident in the bilateral medial occipital cortex (Fig. 1b). The observed MEG alpha- and beta-band suppression, the gamma-band increase and the peak of the GABA_A_ receptor density in the occipital cortex all fell within or showed spatial overlap with the anatomically defined primary visual cortex (Fig. 1c). In addition to the visual regions, there was both modulation of gamma-band activity and a high density of receptors in the left cingulate cortex. In the bilateral V1, the stimulation induced a prominent gamma-band response at around 100–500 ms and in almost the entire evaluated frequency range, peaking at ~300 ms and ~65 Hz (Fig. 1d). The alpha- and beta-band suppressions occurred slightly later, peaking at around 400 ms and 12 Hz (alpha) and at ~350 ms and ~20 Hz (beta). The responses showed substantial variability across subjects, particularly for the gamma-band peak frequency and timing (Fig. 2).

### GABA_A_ receptor density correlates with gamma-band frequency and amplitude in V1

**Figure 3:**
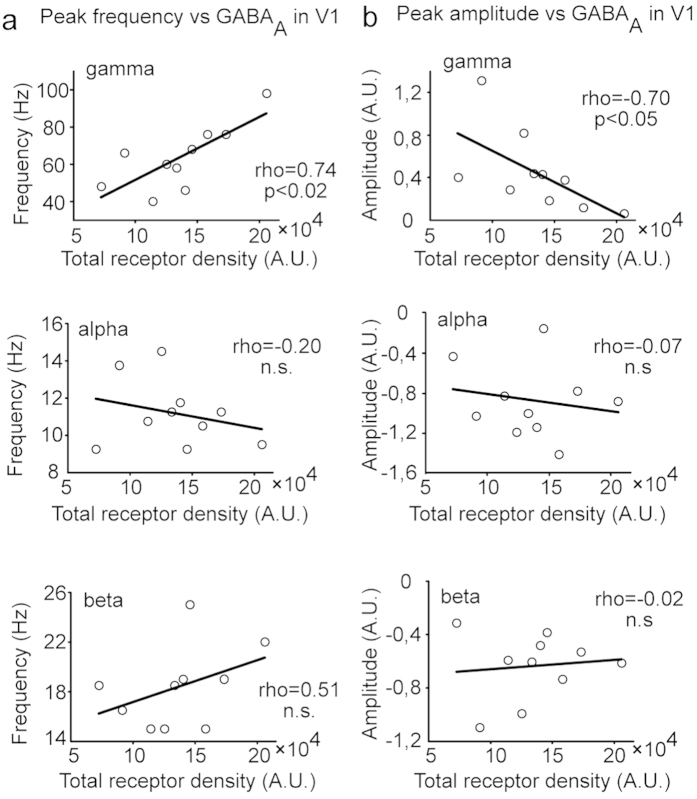
MEG-PET correlation in V1. Correlation between the total GABA_A_ receptor density and the modulation peak (**a**) frequency and (**b**) amplitude in the gamma-, alpha-, and beta-bands.

The correlation analysis between FMZ-PET data and MEG neural response properties in V1 revealed a significantly positive correlation between GABA_A_ receptor density and gamma peak frequency (rho = 0.74, p = 0.014; 95% confidence limits for correlation, CI_95%,_ 0.50 − 0.90; Fig. 3a) and a significantly negative correlation between GABA_A_ receptor density and gamma amplitude (rho = -0.70, p = 0.031; CI_95%_ = −0.86 −0.45; Fig. 3b). The amplitude and frequency of the gamma-band activity were, however, not mutually correlated (rho = -0.47, p = 0.17). By contrast to the gamma-band, the alpha- and beta-bands did not show significant correlations with GABA_A_ receptor density, neither for peak amplitude (alpha, rho = -0.07, p = 0.86; CI_95%_ = −0.44 - 0.33; beta, rho = -0.02, p = 0.97; CI_95%_ = −0.50–0.42) nor for frequency (alpha, rho = -0.20, p = 0.58; CI_95%_ = −0.71 − 0.06; beta, rho = 0.51, p = 0.14; CI_95%_ = 0.27 − 0.80; Fig. 3). Moreover, no significant correlation between peak timing and GABA_A_ receptor density was detected in any of the frequency bands (alpha, rho = 0.23, p = 0.53; beta, rho = 0.6, p = 0.073; gamma, rho = 0.40, p = 0.25).

### Correlation between GABA_A_ receptor density and gamma-band activity is a specific mechanism in V1

**Figure 4:**
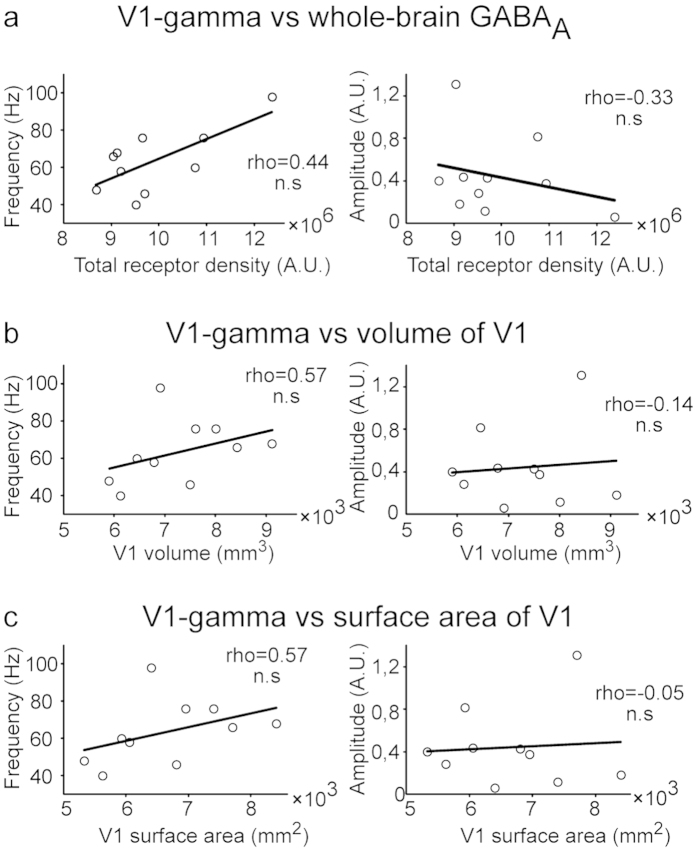
Correlation between V1 gamma-band activity, whole-brain GABA_A_ receptor density and size of V1. (**a**) Correlation between the whole-brain receptor density and the peak frequency and amplitude. (**b**) Correlation between the volume of V1 and the peak frequency and amplitude. (**c**) Correlation between the surface area of V1 and the peak frequency and amplitude.

To assess the robustness and specificity of the correlation observed between GABA_A_ receptor density and V1 gamma oscillation properties, we conducted extensive additional analyses exploring multiple potential confounds. First, to make sure that the observed correlations were specifically linked to the GABAergic properties of the V1 region, we recomputed the correlations replacing V1 receptor density with the overall brain-wide GABA_A_-density. The results (Fig. 4a) demonstrate that the amplitude and frequency properties of V1 gamma-band activity are not correlated with the total GABA_A_-density (amplitude, rho = -0.33, p = 0.35; CI_95%_ = −0.64 − 0.00; frequency, rho = 0.44, p = 0.21; CI_95%_ = 0.13 − 0.71). We also tested whether correlations could arise between the gamma-activity properties and anatomical properties of V1: No significant correlation was detected between the gamma properties and volume (amplitude, rho = -0.14, p = 0.71; frequency, rho = 0.57, p = 0.084 CI_95%_ = 0.32 − 0.74) or surface area (amplitude, rho = -0.05, p = 0.89; frequency, rho = 0.57, p = 0.084; CI_95%_ = 0.20 − 0.71) of V1 (Fig. 4bc). Furthermore, given that previous reports have reported relationships between GABA_A_ receptor and neuronal density^29^ and between gamma frequency and cortical surface area^30^, we also tested whether the observed correlations subsist after regressing out the effect of these parameters. To this end, we replicated the main correlation analyses using PET data from which we first regressed out V1 gray matter density and surface area. The conducted regression strengthened the correlation measured between GABA_A_ receptor density and gamma-band peak frequency (rho = 0.86, p = 0.0013; CI_95%_ = 0.73 − 0.98), whereas the correlation between the receptor density and gamma-band amplitude only showed a trend (rho = -0.56, p = 0.096; CI_95%_ = −0.83 −0.14). The correlations results summarized in Table 1 also show that this tendency was further confirmed using a Bayes factor (BF) analysis, which provided evidence against the null hypothesis of no correlation with gamma frequency (BF = 6.0064; Pearson’s r = 0.75973, p = 0.0108) but no evidence against the null hypothesis when it came to gamma-amplitude (BF = 1.4159, Pearson’s r = -0.61411, p = 0.0589).

To further probe the robustness of our findings, we also computed correlations between V1 gamma oscillation characteristics (peak frequency and amplitude) and the PET-based GABA_A_ receptor density in randomly selected sets of voxels across the brain (either randomly distributed voxels or spatially contiguous random voxel sets). The magnitude of the correlations between the V1 MEG estimates and PET GABA_A_ receptor data from any of the 200 random sets of PET-voxels was systematically lower than the ones obtained with the corresponding V1-PET values. We also explored whether our findings depend on the way the gamma-band modulations were evaluated. By replacing absolute with percent signal change, a significant correlation was still present between the GABA_A_ receptor density and the peak gamma frequency (rho = 0.84, p = 0.0024; CI_95%_ = 0.68 − 0.97); however, the correlation between GABA_A_ receptor density and peak amplitude was no longer significant (rho = -0.34, p = 0.36; CI_95%_ = −0.57 − 0.12). Finally, we found that neither peak frequency nor amplitude of V1 gamma correlated with the subjects’ age (frequency, rho = 0.47, p = 0.17; amplitude, rho = -0.10, p = 0.77).

## Discussion

By combining MEG and Flumazenil-PET measurements the current study reveals that the density of GABA_A_ receptors correlates positively with gamma peak frequency and negatively with gamma amplitude in primary visual cortex. These results support the view that gamma-range neuronal response profiles across individuals are shaped by inhibitory neurotransmission, assessed by GABA_A_ receptor density.

The prominent correlation between GABA_A_ receptors and gamma peak frequency is in-line with modeling work^31^ which predicts that the frequency strongly depends on the balance between AMPA and GABA synaptic currents. This may be explained by the fact that both pyramidal-to-interneuron connections and the pyramidal-to-pyramidal connections tend to decrease population frequency. Our findings thus improve our understanding of the neurochemical mechanisms that determine gamma oscillations in human cortex. Such insights are critical given the importance of precise spike time correlations across networks for synaptic integration and plasticity.

Notably, while the significant positive correlation between GABAergic inhibition and peak V1 gamma frequency agrees with two previous MRS-MEG studies^17^,^18^, the significant negative correlation we observed between GABA_A_ receptor density and gamma amplitude was not observed in these studies. Interestingly, previous results have, in fact, shown that the administration of both alcohol^32^ and lorazepam^33^ may affect both the frequency and amplitude of gamma-band activity in the visual cortex. However, it should be noted that in the present study the statistical significance observed in the gamma amplitude correlation (rho = -0.7, p = 0.031) was weaker than the one we found for gamma frequency (rho = 0.74, p = 0.014). Besides, when regressing out the effect of gray matter density and size of V1, we observed that the gamma frequency correlation was actually enhanced (rho = 0.86, p = 0.0013), whereas the correlation between the receptor density and gamma amplitude only showed a trend to significance (rho = -0.56, p = 0.096). Furthermore, the Bayes factor correlation evidence results also confirm the statistical significance of the gamma-frequency-GABA_A_ but not the gamma-amplitude- GABA_A_ correlation (Table 1).

Previous reports have also suggested that the frequency of gamma-band activity in the visual cortex may depend on the surface area of V1, which, in turn, is linked to the horizontal connectivity and homogeneity within the region^30^. In the present study, correlations between gamma-band peak frequency and V1 surface area or volume did not reach significance (p = 0.084, in both cases). There are two possible explanations for this difference. First, in the study by Schwarzkopf *et al.*^30^, the surface area of V1 was defined using retinotopic mapping, whereas in our study the definition was based on a probabilistic atlas and the curvature of each subject’s cortex. Second, the definition of the surface area of V1 is only a surrogate of the homogeneity of V1, and would thus not necessarily correlate directly with the measured oscillation frequency. Interestingly, other studies have also failed to replicate the findings by Schwarzkopf *et al.*^30^ with respect to the relationship between V1 size and gamma properties^34^. Furthermore, our correlation analyses between FMZ-PET and alpha or beta oscillations provide no evidence for the influence of the GABAergic system on oscillatory amplitude and frequency in V1 at these lower frequencies. Thus, our data indicate that in V1 GABAergic inhibition primarily affects the properties of oscillatory neural responses in the gamma-band.

Exploring the neurochemical basis of neuronal oscillations, and its putative link to GABAergic inhibition in particular is a challenging endeavor^35^. A few recent studies were able, using MEG and MRS measurements, to link the concentration of GABA to the properties of oscillatory activity in the human brain^17^,^18^,^19^,^20^, and, specifically, to the peak frequency of gamma band-activity, in line with predictions from computational models of neural populations^31^. Interestingly, initial reports of correlation between GABA concentration and gamma oscillation frequency in human primary visual cortex^17^ were not replicated by a recent study^24^. The studies differed, among other things, in the number of participants, the type of MRS sequence used and the size of the MRS voxel of interest. Yet, irrespective of the reasons behind the differing results, the diverging findings between these studies but also from other reports^21^,^22^,^23^,^36^ do question the reliability of using MRS as a measure of the GABAergic mechanisms thought to mediate gamma oscillations^24^. MRS measurement of GABAergic inhibition suffers from susceptibility to macromolecule contamination and the need to sample large voxels. In addition, because MRS measures an overall GABA concentration level, it cannot be used to distinguish the contributions of neurotransmitter synaptic pool from the metabolic pool^37^. In fact, the quantification of GABA concentration following selective modulation with Tiagabine suggests that the MRS signal is only weakly affected by synaptic GABA^38^. This highlights the need to examine *in vivo* GABAergic inhibition and the GABA-gamma relationship with alternative techniques^39^. To this end, we chose Flumazenil-PET which allows quantification of GABA_A_ benzodiazepine receptor density, a measure likely to be more directly related to the GABAergic inhibitory mechanisms involved in gamma oscillations; the correlation we found between gamma-frequency and GABA_A_ receptor density in V1 supports the hypothesis that FMZ-PET measurements might indeed be better surrogate for GABAergic inhibition than MRS-based estimates. It should be noted, however, that although our findings were robust to numerous control analyses, they would certainly benefit from replication in a larger cohort. Sample size is a critical limitation in such correlation analyses and may potentially explain the discrepancies previously observed in MRS-based studies on the same question^17^,^24^.

In a sense, our results are consistent with both the Muthukumaraswamy *et al.*^17^ and the Cousijn *et al.*^24^ studies: The significant GABA-gamma frequency correlation replicates and extends results of the former study, and the fact that it has been unraveled using a technique more selective to GABAergic inhibition is in line with conclusions of the latter study. Moreover, discrepancies between MRS-based GABA-concentration and FMZ-PET-based GABA_A_ receptor density measures are to be expected. It has been shown that vigabatrin may change the tissue GABA levels without an associated change in the benzodiazepine downregulation^40^, and TMS-based estimates of synaptic GABA_A_ and GABA_B_ activity do not necessarily correlate with the MRS-based concentration measures^41^,^42^,^43^.

Nevertheless, it is important to note that the claim made here about the potential higher suitability of FMZ-PET compared to MRS, only relates to the specific question of linking gamma oscillation properties to GABAergic inhibition. MRS-mediated GABA concentration measures provide highly relevant findings linking GABA levels to behavior. For instance, GABA concentration in frontal eye fields (FEF) predicts saccade distractibility^44^ whereas its concentration in supplementary motor area (SMA) is correlated with responsiveness of subconscious motor mechanisms^45^. Whether such explorations would benefit in any way from measuring GABA_A_ receptor densities is an open question.

Finally, it is noteworthy that although FMZ-PET provides an assessment of GABA_A_ receptor density rather than total GABA, neither FMZ-PET nor MRS-derived measures provide a direct quantification of inhibition strength. Flumazenil cannot be used to isolate a specific synaptic GABA-benzodiazepine receptor signal: The ^[11C]^Flumazenil radioligand binds with similar affinity to synaptic (α1-α3) and to extrasynaptic (α5) subunits of GABA-benzodiazepine receptors. In contrast, recent findings indicate that the inverse agonist GABA-benzodiazepine receptor PET tracer ^[11C]^Ro15-4513 can be used to quantify specific synaptic (alpha-1 subtype) signals^46^ and can detect acute increases in synaptic alpha-1 GABA^37^. Combining data from ^[11C]^Ro15-4513 PET with MEG measurements could help fine-tune the relationship between GABAergic inhibition and gamma oscillations. Moreover, it should be noted that at present it is difficult to associate our findings with the extensive modeling, animal and imaging work that shows that increased GABAergic efficacy (as measured by the inhibitory postsynaptic current decay times) leads decreased gamma frequencies^32^,^33^,^47^,^48^,^49^. Our results suggest that the PET based GABA_A_ receptor density measurement can serve as a proxy for the total inhibition strength that has been suggested to also affect the gamma frequency^31^. It is, however, unclear how the GABA_A_ receptor density links to GABAergic efficacy and the various network kinematics properties that have been shown to influence the frequency of gamma oscillations^8^.

In conclusion, the present results show a prominent correlation between PET based measurements of GABA_A_ receptor density and MEG based recordings of gamma-band oscillations in primary visual cortex. These results provide the first evidence in humans for a direct link between gamma oscillation properties and inhibitory neurotransmission indexed by GABA_A_ receptor density. Moreover, our findings also suggest that Flumazenil based PET-GABA_A_ estimates provide a spatially specific measure of GABAergic inhibition that, in combination with MEG measurements, could help elucidate the mechanisms that determine the properties of gamma-band oscillations and their variability across individuals in health and disease.

## Methods

### Subjects and experimental design

Thirteen healthy, native French-speaking subjects participated in the study. For one subject, the MEG recording failed due to technical reasons, and a second subject did not participate in the PET recording. In addition, the MEG data of a third subject had to be discarded due to excessive blinking. Both PET and MEG data of sufficient quality were thus recorded from 10 subjects (1 female, 9 males; age 19–29 years, mean 24 years). Informed consent was obtained from all subjects, in agreement with the prior approval of the Institutional Review Board and by the National French Science Ethical Committee (CPPRB). All methods were conducted in accordance with the CPPRB guidelines. The MEG experiment consisted of a classical working memory task of visually presented letters, with 3 s intervals between consecutive letters. Each letter was shown for 300 ms. The paradigm comprised three different memory load conditions (1-back, 2-back and 3-back tasks), and the subjects indicated with a button press, after each letter, whether the letter matched or did not match the appropriate preceding letter. Here, we focus on the initial average visual response across the three tasks for the non-target trials; we excluded the target-trials where the subjects’ behavior would have been markedly different with respect to the non-target trials. These trials and the early time-window contain the neural responses that reflect the perception and encoding of the presented letters to memory. The PET data used to determine the GABA_A_ receptor density were collected while the subjects were resting eyes-closed in the scanner.

### MEG, PET and MRI data collection

All data (MEG, PET and MRI) were collected from the same participants at the CERMEP imaging centre (Lyon, France). The MEG data were recorded in a magnetically shielded room using a 275-channel CTF whole-head system. The signals were band-pass filtered at 0.016-150 Hz and sampled at 600 Hz. The PET data were recorded with a Siemens HR + camera, and ^[11C]^flumazenil (FMZ), an agent that binds to benzodiazepine (BZD) receptor, was used. FMZ (RO15-1788) was labeled with 11C, using the methylation process^50^. A dynamic 3D acquisition was applied providing 12 consecutive frames of 63 contiguous 2.42 mm thick slices, with an isotropic spatial resolution near 5 mm^3^ FWHM (full width at half-maximum). A 68Ge transmission scan was used to measure the attenuation correction. A partial saturation protocol consisting of a single intravenous injection of a mixture of 5 mCi of ^[11C]^FMZ and 0.01 mg/kg of unlabeled FMZ was used, followed by acquisition of the emission data for 55 minutes. This single injection allowed the calculation of B′max parametric images, i.e., estimates of receptor density, without arterial blood sampling^51^. Anatomical MRIs were collected with a 1.5 Tesla scanner (Siemens Sonata Maestro Class).

### MEG data analysis

Neural activity estimates were obtained from the MEG signals with event-related Dynamic Imaging of Coherent Sources (erDICS)^52^, a beamforming technique in the time-frequency domain. In erDICS, the cortical-level beamforming estimates are based on the sensor-level time-dependent cross-spectral density matrix (CSD), computed using Morlet wavelets^53^. Here, wavelets of width 7 were used to calculate the CSD in the range from 200 ms before stimulus onset to 800 ms post stimulus at 17 ms intervals; the width parameter defines both the spectral bandwidth and duration of the of the wavelets. The estimation of the time-frequency data was conducted by constructing wavelets at different frequency intervals at the different frequency ranges; the range from 4 to 15 Hz was sampled with 0.25 Hz resolution, whereas the range from 15 to 30 Hz and 36 to 104 Hz were sampled with 0.5 and 2 Hz resolutions, respectively. Trials in which the amplitude of either the vertical or the horizontal electro-oculogram exceeded 150 μV were rejected. In addition, the MEG data were examined visually and data segments that contained artifacts were excluded from the analysis. The number of accepted trials varied between 151–254 across subjects. The beamforming estimates of cortical oscillatory power levels at different frequencies were computed, using a spherical head model, at each time-instance, as an average of the time-bin itself and six preceding and following time-bins; the baseline activity level per frequency bin was estimated as the average power in the −200 to 0 ms baseline interval (total of 13 time-bins as well). For frequencies up to 30 Hz, the estimates were computed for individual frequency bins, whereas in the interval of 40–100 Hz, we averaged the data across an 8 Hz window at each bin to reduce the effects of noise (e.g., estimate of activity of 40 Hz was calculated based on the average CSD at the interval of 36–44 Hz). The general effects of the stimulation were evaluated at the group-level (paired t-test, p < 0.005, uncorrected) by comparing the average activity in the alpha (8–13 Hz), beta (15–25 Hz) and high-gamma band (60-90 Hz) between the pre-stimulus (-200 to 0 ms) and the post-stimulus (0 to 600 ms) time-windows. For visualization, the results were projected to the surface of the brain.

### PET data analysis

Static PET images were obtained by summing frames 8–12 from the dynamic imaging (corresponding to the acquisition period from 20 to 55 min post-injection). These static images of the PET volumes were realigned to the anatomical MRIs. Ten millimeter circular ROIs were placed over the midportion of the pons, a suitable reference region for the calculation of the non-specific FMZ binding^51^; the reference encompassed 7–9 consecutive MRI slices displaying that structure. These ROIs were then transferred onto the corresponding FMZ-PET slices. An additional circular ROI with 15 mm diameter was placed over the occipital cortex which commonly displays a high concentration of BZD receptors^54^. A partial-saturation model, based on a Scatchard plot, was then used to obtain the B′max parametric images^55^. In this model, the free ligand concentration is estimated in the pons, whereas the range of the bound ligand concentration is evaluated in the occipital cortex. Using this approach we obtained, for each subject, a set of 63 contiguous 2.42 mm thick parametric images of BZD receptor B′max. Each individual’s PET data were smoothed using a 15 × 15 × 21 mm full width at half maximum Gaussian kernel; the level of smoothing was chosen to achieve similar resolution to the MEG estimates of neural activity^56^.

### MEG-PET correlation analysis in the primary visual cortex

The MEG estimates were computed separately in each voxel of a grid that covered the gray matter surface of the brain. An equivalent spatial sampling across subjects was achieved by first forming a volumetric grid with 6-mm sampling in the template brain. A grid point was included in the analysis when it was within 1.5 cm of the brain surface. The template brain grid, consisting of 3922 points, was transformed, via an elastic transformation (implemented in SPM8; Wellcome Trust Centre for Neuroimaging), to each individual’s anatomy. The MEG beamforming estimates were calculated directly at these individual-level grid points. The PET-data were coregistered with each individual’s anatomical MRI in SPM8.

The MEG-PET correlation analysis was conducted in the bilateral primary visual cortex (V1). The anatomical definition of V1 (see Figs 1c, 2) was based on the automatic segmentation and parcellation procedure in Freesurfer 5.3^57^; in the procedure, the bilateral V1 is defined based on a probabilistic atlas and subject-specific cortical folding patterns^58^. Following the segmentation of the gray matter surface and the determination of the V1 labels (left/right) in the surface space, the labels were transformed into volumetric data and the MR-voxels corresponding to the V1-gray matter surfaces were identified. With regard to the MEG data, we determined, for each subject, the grid points that belonged to V1, and collected the MEG estimates across these grid points. The amplitudes of each subject’s data were normalized by the standard deviation in all V1 grid points across all frequency bins in the baseline time-window; the normalized estimates were used to eliminate the effects of head and brain geometry on the amplitude estimates. This normalization was conducted separately for the low (4 to 30 Hz) and high-frequency (40-100 Hz) bands. For the PET data, we determined for each V1 MR-voxel the closest corresponding PET-voxel, and defined the unique set of these V1-PET voxels. The MEG responses were computed by subtracting the average activity during the baseline time-window from the post-stimulus time-bins, separately at each frequency; the MEG responses used in the MEG-PET correlation analysis were obtained by averaging the MEG responses across the V1 grid points. For each subject, we identified the peak frequency, timing and amplitude of the MEG responses, separately for the alpha- (7-14 Hz), beta- (15-30 Hz), and gamma-bands (40-100 Hz). The peak frequency was identified as the frequency showing the largest modulation between 0 and 600 ms; the peak timing was defined as the time-instance between 0 and 600 ms showing the largest modulation of activity in each band (alpha, beta, gamma); the peak amplitude was determined at the time-frequency bin corresponding to the peak frequency and time-instance. The amplitude measures are reported in arbitrary units as they have been normalized by the standard deviation across frequencies and V1 grid-points. As the GABA_A_ estimate we used the total GABA_A_ receptor density in the unique set of V1-PET voxels. The correlation between the PET-GABA_A_ estimates and the MEG estimates were computed using Spearman’s rho.

We also evaluated the possibility of finding correlations between the V1-specific MEG estimates and PET-GABA_A_ estimates in random voxel sets outside the visual cortex (selected from among voxels that were more than 4 cm from the primary visual cortex). Here, we selected 100 sets of fully random voxels and 100 sets of random contiguous voxels (clusters); for both set-types, an equal number of voxels to voxels in each subjects’ V1 were selected. We also investigated whether it would matter if one quantifies the modulations as an absolute or percent signal change between the post-stimulus and baseline time-windows. In addition to the MEG-PET correlation analysis, we also evaluated the correlation between the size of V1 (volume and surface area) and the MEG responses. Moreover, because FMZ-PET based measures of GABA_A_ receptor density have been used as a surrogate marker for neuronal density^29^, it is possible that any observed relationship between GABA_A_ density estimates and rhythmic activity could be due to inter-subject differences in neuronal volume of V1. Moreover, previous reports have suggested that the gamma-band frequency is dependent on the surface area of V1^30^. To account for the contribution of these possible confounding factors, we applied linear regression to remove the effects of V1 surface area and gray matter density from the PET based GABA-A estimates and ran the main correlation analyses over again. Note that the gray matter density in V1 was determined by first computing the proportion of gray matter for each PET voxel and by averaging these values across V1. Unless mentioned otherwise, for all correlations analyses reported here between GABA_A_ receptor density on one hand and oscillatory amplitude or frequency on the other, across the population, we used uncorrected p-values.

Finally, in addition to Spearman’s rank correlation analysis, we also computed Pearson’s correlation coefficient and a Bayes factor (BF) based on the approach described by Wetzels and Wagenmakers^59^. BF summarizes the ratio of evidence for a correlation of any kind against the evidence for no correlation (the null hypothesis). Despite the lack of clear-cut categorical boundaries, a BF < 0.3 is taken as an indication in favor of and a BF > 3 as strong evidence against the null hypothesis^60^.

As with low number of subjects the correlation estimates may be influenced by outliers in the data, we computed the 95% confidence limits for the correlation estimates using bootstrapping. In the approach we left out data pairs for 2 random subjects 200 times, and computed the correlation and p-values for the re-sampled data. From the obtained distribution of 200 correlation-values we calculated the 95% upper and lower confidence limits. This procedure was applied to all main correlation analyses.

## Additional Information

**How to cite this article**: Kujala, J. *et al.* Gamma oscillations in V1 are correlated with GABA_A_ receptor density: A multi-modal MEG and Flumazenil-PET study. *Sci. Rep.* **5**, 16347; doi: 10.1038/srep16347 (2015).

## Figures and Tables

**Table 1:** Correlation between GABA_A_ receptor density and gamma-band activity in V1.

**Correlation**	**Spearman’s rho**	**Pearson’s r**	**Bayes Factor (BF)**
Gamma_frequency_ -GABA_A_	0.74 (p = 0.014)	0.76 (p = 0.011)	6.00
Gamma_frequency_ -GABA_A (regressed)_	0.86 (p = 0.0013)	0.91, (p = 0.0002)	163.25
Gamma_amplitude_ -GABA_A_	−0.70 (p = 0.031)	−0.61 (p = 0.056)	1.42
Gamma_amplitude_ -GABA_A (regressed)_	−0.56 (p = 0.096)	−0.49 (p = 0.15)	0.68

Overview of results and statistical significance of the correlation analyses between GABA_A_ receptor density and gamma frequency and amplitude in V1, measured respectively with FMZ-PET and MEG. Four correlations were investigated using three distinct correlation measures (Spearman, Pearson and a Bayes factor). The four correlations examined (left most column) are the correlations between either gamma frequency or gamma amplitude with GABA_A_ receptor density either with (GABA_A (regressed)_) or without (GABA_A_) regression step.
